# Comparative evaluation of thrombocytopenia in adult patients receiving linezolid or glycopeptides in a respiratory intensive care unit

**DOI:** 10.3892/etm.2013.1437

**Published:** 2013-12-04

**Authors:** ZHAORUI ZHANG, ZHIXIN LIANG, HUAIDONG LI, LIANG’AN CHEN, DANYANG SHE

**Affiliations:** 1 Department of Respiratory Medicine, Chinese PLA General Hospital, Haidian, Beijing, P.R. China; 2 Department of Respiratory Disease, The 88^th^ Hospital of Chinese PLA, Tai’an, Shandong, P.R. China

**Keywords:** thrombocytopenia, linezolid, glycopeptides

## Abstract

Linezolid is an oxazolidinone antibiotic agent, active against gram-positive bacteria that are resistant to traditional antibiotics, including glycopeptides. Linezolid is generally well tolerated, but has been associated with hematologic adverse effects such as thrombocytopenia. The primary objective of this study was to compare the incidence of thrombocytopenia between patients receiving linezolid or glycopeptides in different age groups. The secondary objective was to assess the association between the time-to-event and occurrence of thrombocytopenia. This retrospective study reviewed the medical records of patients who were treated with linezolid or glycopeptides (vancomycin or teicoplanin) between January 2010 and June 2013 in a respiratory intensive care unit. Data were extracted from the patients’ electronic medical records, which were obtained from a central database in the hospital, and multivariate analyses were performed. In total, the study included 225 patients who received linezolid or glycopeptides. The cumulative probability of thrombocytopenia was higher in the patients receiving linezolid than in those receiving glycopeptides (P<0.05), however the cumulative probability of thrombocytopenia did not differ significantly between patients receiving linezolid or glycopeptides in the subgroup whose age was <65 years (P>0.05). With a treatment duration of ≥7 days, the incidence of thrombocytopenia and the mean platelet count reduction in the patients receiving linezolid was significantly higher than in those receiving glycopeptides (P<0.05). No significant difference was identified in the mean platelet counts between the patients receiving linezolid and those receiving glycopeptides. In conclusion, it was identified that patients in a respiratory intensive care unit, aged ≥65 years or with a treatment duration of ≥7 days who were treated with linezolid were more likely to develop thrombocytopenia than patients of the same subgroup who were treated with glycopeptides.

## Introduction

Gram-positive infections are associated with a high incidence of morbidity and mortality in respiratory intensive care units (ICUs) in China (1). The mortality rate for methicillin-resistant *Staphylococcus aureus* (MRSA) pneumonia has been reported to range from 33 to 55% in certain Asian countries, including Japan, Taiwan, Singapore, China and Korea (2,3). In a number of studies, the rate of thrombocytopenia identified in seriously ill patients, particularly those in ICUs, has ranged from 19 to 63% (4–11). Thrombocytopenia is strongly associated with poor outcome, illness severity, the development of organ failure, the length of hospital stay and mortality (5,7,10,12). A common cause of acquired thrombocytopenia is the use of drugs, including heparin, glycoprotein and antibiotics, in the ICU (13). Linezolid, the first oxazolidinone antibiotic agent, has demonstrated antibacterial activity against gram-positive bacteria, including MRSA and vancomycin-resistant enterococci. Although linezolid is safe, it has been demonstrated to be associated with hematologic effects, including thrombocytopenia, anemia and neutropenia. Thrombocytopenia is the most frequent adverse effect associated with linezolid (14,15). However, only a limited number of well-designed studies have compared the incidence of thrombocytopenia in patients treated with linezolid with that in patients treated with other anti-gram-positive agents (16–19).

Since 2007, linezolid has been available in China and thrombocytopenia associated with linezolid has been reported (20). However, the incidence of thrombocytopenia associated with linezolid varies in different countries, such as Japan and United States (21–23). The incidence of thrombocytopenia and time-to-event compared with comparators in a large sample of Chinese patients in an ICU has, to the best of our knowledge, not been reported to date.

The primary purpose of this investigation was to compare the incidence of the thrombocytopenia associated with the use of linezolid and glycopeptides in different age groups. The secondary purpose was to conduct a time-to-event analysis between patients receiving linezolid and glycopeptides. In order to assess this, the mean number of platelets and the reduction in the number of platelets were compared between patients receiving linezolid and those receiving glycopeptides.

## Patients and methods

### Patients

This study was conducted on patients in the respiratory ICU of the Chinese PLA General Hospital (Beijing, China). Patients who received linezolid (Pfizer Inc., New York, NY, USA; 1,200 mg as a 600-mg dose every 12 h), vancomycin (Eli Lilly and Company, Indianapolis, IN, USA) or teicoplanin (Sanofi-Aventis Ltd., Paris, France) via intravenous administration were included in the study. The daily dosage of the vancomycin or teicoplanin was according to the manufacturer’s instructions. The vancomycin dosage was 15 mg/kg/day for patients with normal renal function and was adjusted according to creatinine clearance. Teicoplanin was used at a dose of 12 mg/kg/day for patients with normal renal function and was also adjusted according to the creatinine clearance. The data were collected from the electronic medical records in the hospital’s central database of patients who were treated between January 1, 2010 and June 30, 2013.

The inclusion criteria were as follows: i) Patients with pneumonia who received linezolid or vancomycin or teicoplanin for ≥5 days in the respiratory ICU for the treatment or prophylaxis of pulmonary gram-positive infections; ii) the baseline number of platelets was available; and iii) the platelets were examined every day from the start to the end of the treatment.

The exclusion criteria were as follows: i) age <18 years; ii) patients with a blood system or platelet number reduction associated with an original disease, including leukemia, myelodysplasia, aplastic anemia and tumors being treated using chemotherapy; iii) patients who had experienced platelet transfusion during the period when using linezolid or glycopeptides; and iv) an abnormal platelet count (<100×10^9^/l or >400×10^9^/l) prior to therapy.

### Data Extraction

Examination data and clinical notes were extracted from electronic medical records. Medical charts/records without patient names and/or identification numbers were stored in a Microsoft Excel file. Data analyses performed by one doctor were reviewed and confirmed by another doctor to ensure accuracy. As this was a retrospective study, informed consent was waived.

Data included demographics, comorbidities, hospitalization history, disease severity, microbiological data and laboratory data.

Demographic characteristics included age, gender, height, body weight, acute physiology and chronic health evaluation-II (APACHE-II) score, length of hospital stay, treatment duration, renal failure, outcome, mechanical ventilation and infection type (hospital-acquired or community-acquired). Comorbidities included heart failure, respiratory failure, renal failure, chronic obstructive disease, diabetes mellitus, hypertension, hepatic dysfunction, transplanted organ, active malignancy and cancer. Laboratory data included creatinine levels, baseline platelet count and blood urea nitrogen. Microbiological data included the type of organism.

### Definitions

Thrombocytopenia was defined as a platelet count of <100×10^9^/l. The reduction of the platelet count was calculated as follows: Reduction in platelet count = platelet count_baseline_-platelet count_value_.

### Statistical analysis

Statistical analysis was performed using commercially available software (SPSS software, version 15.0, SPSS, Inc., Chicago, IL, USA). The Student’s t-test (normal-distribution) or Mann-Whitney U test (non-normal distribution) was used to compare continuous variables. Categorical variables were compared using χ^2^ test. The multi compression was performed by one-way analysis of variance. For the analyses where the expected cell frequency was low, Fisher’s exact test was utilized. P<0.05 was considered to indicate a statistically significant difference. The hazard ratio with the 95% confidence interval was calculated for each significant variable. Time-to-event analyses were performed by computing Kaplan-Meier estimators (product-limit method) for each case of thrombocytopenia under study. Survival distributions were compared using the log-rank test.

## Results

### Patient data

Of 225 patients enrolled, 122 received linezolid and 103 received glycopeptides (vancomycin or teicoplanin) for ≥5 days. The demographics and clinical characteristics of the study population are displayed in Table I. The demographic characteristics of the patients were similar in the two treatment groups. However, the mean body weight of the glycopeptide group was higher than that of the linezolid group (62.4±13.2 kg for the linezolid group vs. 66.7±13.0 kg for the glycopeptide group) (P=0.01). The incidence of renal failure in the linezolid group (24.6%) was significantly higher than the incidence of renal failure in the glycopeptide group (4.9%) (P<0.01).

**Figure 1:**
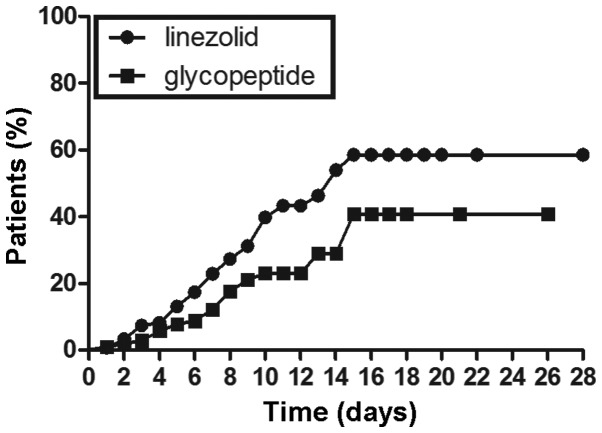
Kaplan-Meier plot of the probability of the number of platelets being <100×10^9^/l. Log rank test P<0.001.

**Figure 2:**
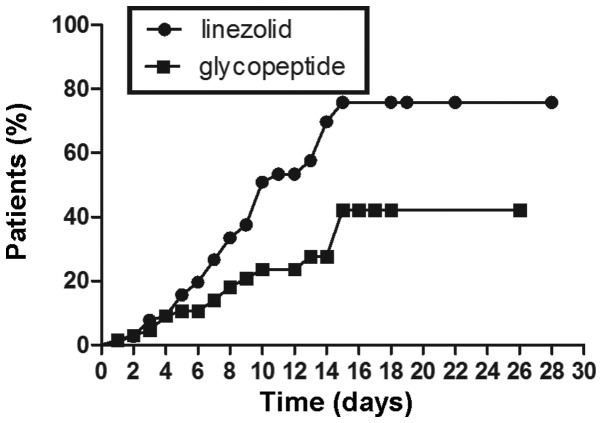
Kaplan-Meier plot of the probability of the number of platelets being <100×10^9^/l in patients aged ≥65 years. Log rank test P=0.002.

**Figure 3:**
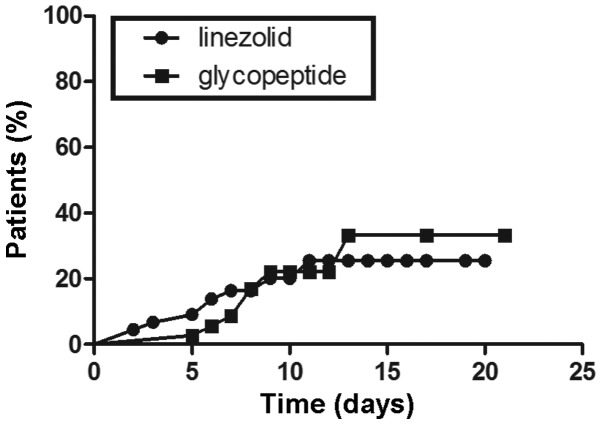
Kaplan-Meier plot of the probability of the number of platelets being <100×10^9^/l in patients aged <65 years. Log rank test P=0.884.

Kaplan-Meier plots of the percentage of patients with thrombocytopenia are displayed in Figs. 1–3. The cumulative probability of thrombocytopenia was significantly higher in the linezolid group than in the glycopeptide group by the log rank test P<0.01 (Fig. 1). Further, a subgroup analysis by age was conducted. In the age ≥65 years subgroup, a similar result to the result of the whole group was obtained. Linezolid was associated with a higher incidence of thrombocytopenia than glycopeptides were, with log-rank test P<0.05 (Fig. 2). However, in the patients aged <65 years subgroup, no significant differences were identified in the probability of thrombocytopenia between the two groups (P>0.05; Fig. 3).

The incidence of thrombocytopenia in the linezolid and glycopeptide groups is displayed in Table II. No significant differences were identified in the incidence of thrombocytopenia between patients receiving linezolid or glycopeptides in first six days of usage of the drug. On day seven, the incidence of thrombocytopenia began to differ, with a significant difference between the two groups and a 95% CI of 1.03–4.51 (P=0.04). On days 7–14, the incidence of thrombocytopenia in patients treated with linezolid remained higher than that in the patients treated with glycopeptides (P<0.05).

The mean number of platelets and the mean reduction in the number of platelets was also analyzed between the two groups. After 12 days, the number of patients in the linezolid group was <30; therefore, the mean number of platelets and reduction in the number of platelets was not compared after 12 days of treatment. The mean numbers of platelets in the linezolid and glycopeptide groups at different time-points are displayed in Table III. The mean platelet counts were similar between patients who received linezolid or glycopeptides during the treatment (P>0.05). Furthermore, the mean reductions in the numbers of platelets were calculated and analyzed. The reductions in the numbers of platelets at different time-points are displayed in Table IV. No significant differences were identified in the mean reductions in the number of platelets between the two groups on days 1–6 (P>0.05). On days 7–12, the mean reductions in the numbers of platelets were significantly higher in the linezolid group than in the glycopeptide group (P<0.05).

## Discussion

Thrombocytopenia is a common adverse event associated with the administration of linezolid. This retrospective study analyzed the thrombocytopenia induced by the use of linezolid or glycopeptides in critically ill patients with documented or suspected gram-positive bacterial infections. With regard to the renal toxicity of glycopeptides, a previous study identified that the incidence of renal failure was higher in patients treated with linezolid than in those treated with glycopeptides (24). Currently, there are no uniform diagnostic criteria for linezolid-induced thrombocytopenia. Thrombocytopenia maybe defined as a platelet count <100×10^9^/l (criterion 1) or a 25% reduction from baseline count (criterion 2) (21). For patients with a normal baseline number of platelets, thrombocytopenia is usually defined as a platelet count of <100×10^9^/l (21). Criterion 2 is used for small reductions in platelet counts, usually in patients with baseline numbers of platelets that are less than the lower limit of the normal range (15). In the present study, patients with abnormal baseline numbers of platelets were excluded, so criterion 1 was used as the definition of thrombocytopenia. The Kaplan-Meier curves suggest that linezolid was associated with thrombocytopenia more frequently than glycopeptide therapy was (P<0.05). Age and long periods of drug administration are known independent risk factors for thrombocytopenia in adult Chinese patients (25). The cutoff point for risk factors has been determined by well-known human pathologic classifications, such as an age of ≥65 years (elderly group), rather than the analysis of clinical data. In the present study, a subgroup analysis was conducted regarding the cumulative probability of thrombocytopenia in the age <65 years and age ≥65 years subgroups. In the age ≥65 years subgroup, the result was consistent with the result of the whole group. The cumulative incidence of thrombocytopenia in patients receiving linezolid was significantly higher than that of the patients receiving glycopeptides in the subgroup age ≥65 years (P<0.05). However, in the subgroup age <65 years, no significant differences in the cumulative incidence of thrombocytopenia were identified between the patients receiving linezolid and those receiving glycopeptides (P>0.05). These findings are consistent with those of previous studies (26,27). Falagas *et al* conducted a meta-analysis which included 12 randomized controlled trials (RCTs) involving 6,093 patients and identified that thrombocytopenia was recorded more commonly in patients receiving linezolid [odds ratio=11.72 (95% CI, 3.66–37.57)] than in patients receiving glycopeptide or β-lactams (26). Kalil *et al* conducted a meta-analysis including nine RCTs and demonstrated that the risks of thrombocytopenia (95% CI, 1.30–2.87; p=0.001) were higher with the use of linezolid than with the use of glycopeptides (27). However, certain studies have observed no significant difference in the incidence of thrombocytopenia between patients who received linezolid and those who received glycopeptides (19,28). An *et al* conducted a meta-analysis of 12 RCTs and the results indicated that there were no significant differences in the incidence of thrombocytopenia between linezolid- and glycopeptide-treated groups (29). In an evaluation of thrombocytopenia risk (defined as ≤150,000 platelets/mm^3^) in 686 patients with nosocomial pneumonia enrolled in two randomized trials, no statistically significant differences were identified in the incidence of thrombocytopenia between linezolid recipients (6.4%) and vancomycin recipients (7.7%) (22). None of these studies conducted a subgroup analysis by age; to the best of our knowledge, the present study is the first trial that has compared the cumulative incidence of thrombocytopenia between patients treated with linezolid and glycopeptides in different age groups. Age has been shown to be an independent risk factor of linezolid-induced thrombocytopenia (22). The functions of organs degenerate and metabolism slows in elderly patients; therefore, drugs more readily accumulate in elderly patients. Hiraki *et al* demonstrated a positive correlation between the incidence of thrombocytopenia and the plasma concentration of linezolid (30). The slowing of the metabolism may be one of the mechanisms responsible for the increased frequency of thrombocytopenia in elderly patients.

Furthermore, in the present study a time-to-event analysis of the incidence of thrombocytopenia was conducted. The risk of thrombocytopenia increases over time, so it is important to determine a mean time at which the cumulative percentage of patients with low platelet counts begins to diverge between linezolid and a comparator. Rubinstein *et al* analyzed seven comparator-controlled clinical trials and identified that with longer treatment durations (>14 days) the cumulative percentage of patients with thrombocytopenia was slightly higher in the linezolid group than in the comparator group (18). However Orrick *et al* proposed that the risk for thrombocytopenia may occur earlier than the 14-day frame and demonstrated that the median duration of therapy for patients who developed thrombocytopenia was 12 days (31). These two studies did not include critically ill patients in respiratory ICUs. The present study identified that the mean duration of therapy when the incidence of thrombocytopenia and the mean reduction in the number of platelets were higher for the linezolid group was 7 days in the critically ill patients in the respiratory ICU. The median duration of therapy for patients who developed thrombocytopenia in the present study was shorter than that in previous studies (18,31). Respiratory tract infections, the severity of the illness and APACHE-II scores are reported to be independent risk factors of thrombocytopenia induced by linezolid (23). The patients enrolled in the present study were critically ill in a respiratory ICU with pneumonia and had high APACHE-II scores, which may have increased the incidence of thrombocytopenia and shortened the mean treatment duration prior to the occurrence of thrombocytopenia.

In contrast with other studies, the design of the present study and its setting provided several strengths. The patients included in the study were all from a respiratory ICU and had respiratory infections. A subgroup analysis was conducted between the linezolid and glycopeptide-treated patients by the age of 65 years. All the platelet counts were recorded from the start to the end of the therapy and the patients with missed platelet counts were excluded from the study. Based on the initial data, it was possible to calculate the mean platelet count and the reduction in the number of platelets for every day until the end of the therapy.

Several considerations should be noted when interpreting the results: i) This was a retrospective analysis so there were no randomizations between the two groups; ii) this study focused on patients receiving therapy at the ICU of the Chinese PLA General Hospital and the choice of study population may limit the ability to generalize these results to other populations; iii) low pretreatment platelet count, low body weight, low serum albumin concentration, long-term drug administration, advanced age and renal insufficiency are all reported to be risk factors for linezolid-induced thrombocytopenia (32), however, only a subgroup analysis by age was conducted and the risk of thrombocytopenia according to other risk factors was not compared.

Overall, the present study concluded that linezolid treatment was associated with a higher incidence of thrombocytopenia compared with glycopeptide treatment, mainly in elderly patients aged ≥65 years or with a treatment duration of ≥7 days. Based on the data, linezolid should be used more carefully in patients whose age is ≥65 years or treatment duration is ≥7 days and the platelet count should be monitored during the treatment. However, a stricter prospective study is required to support these findings.

## Figures and Tables

**Table I:** Clinical and demographic characteristics of patients receiving linezolid or glycopeptide therapy.

Matching variables	Linezolid group (n=122)	Glycopeptide group (n=103)	P-value
Age (n,%)	65.9±18.2	69.3±16.1	0.14
<65 years	44 (36.1)	37 (35.9)	0.98
≥65 years	78 (63.9)	66 (64.1)	
Gender			
Male	78 (63.9)	69 (67.0)	0.63
Female	44 (36.1)	34 (33.0)	
Mean height (cm)	167.3±7.2	167.6±7.3	0.73
Mean body weight (kg)	62.4±13.2	66.7±13.0	0.01
Treatment duration (days)			
Mean ± SD	9.9±3.9	10.5±4.6	0.24
Range	5–28	5–28	
Mean length of hospital stay (days)	27.7±17.2	30.6±20.0	0.26
APACHE-II score	17.6±7.2	15.7±6.7	0.06
Baseline creatinine level (μmol/l)	95.6±110.9	98.6±124.4	0.85
Baseline BUN (μmol/l)	8.4±6.5	8.7±7.1	0.69
Mechanical ventilation	90 (73.8)	81 (78.6)	0.39
Infection type			
Community-acquired	15 (12.3)	12 (11.7)	0.88
Hospital-acquired	107 (87.7)	91 (88.3)	
Number of comorbities			>0.05
0	17 (13.9)	18 (17.5)	
1	30 (24.6)	26 (25.2)	
2	34 (27.9)	30 (29.1)	
3	38 (31.1)	35 (34.0)	
≥4	33 (27.0)	24 (23.3)	
Renal failure	30 (24.6)	5 (4.9)	<0.01
Death	30 (24.6)	25 (24.3)	0.96
Organism			
MRSA	51 (41.8)	45 (43.7)	0.78
MSSA	22 (18.0)	18 (17.5)	0.91
*Enterococcus* spp.	27 (22.1)	22 (21.4)	0.89
Others	22 (18.0)	15 (14.6)	0.48

Data presented as n (%) or mean ± SD (range). APACHE-II, acute physiology and chronic health evaluation-II; BUN, blood urea nitrogen; MRSA, methicillin-resistant *Staphylococcus aureus*; MSSA, methicillin-sensitive *Staphylococcus aureus*.

**Table II:** Risk of adverse platelet outcome (platelet count <100×10^9^/l) between the linezolid and glycopeptide groups at different time-points.

Time (days)	Linezolid group	Glycopeptide group	Risk ratio	95% CI	P-value
Overall	46 (37.7)	23 (22.3)	2.10	1.17–3.81	0.01
1	1 (0.8)	1 (1.0)	0.84	0.05–13.65	1.00
2	4 (3.3)	2 (1.9)	1.71	0.31–9.54	0.69
3	9 (7.4)	3 (2.9)	2.65	0.70–10.08	0.23
4	10 (8.2)	6 (5.8)	1.43	0.51–4.12	0.49
5	16 (13.1)	8 (7.8)	1.79	0.73–4.37	0.19
6	21 (17.2)	9 (8.7)	2.17	0.95–4.97	0.06
7	27 (22.1)	12 (11.7)	2.16	1.03–4.51	0.04
8	31 (25.4)	14 (13.6)	2.12	1.05–4.25	0.03
9	34 (27.9)	15 (14.6)	2.18	1.11–4.31	0.02
10	40 (32.8)	19 (18.4)	2.16	1.15–4.03	0.02
11	40 (32.8)	19 (18.4)	2.16	1.15–4.03	0.02
12	42 (34.4)	19 (18.4)	2.32	1.25–4.33	0.01

All data are presented as n (%). CI, confidence interval.

**Table III:** Mean platelet counts at different time-points.

Time (days)	Patients (n)	Linezolid group (mean ± SD)	Patients (n)	Glycopeptide group (mean ± SD)	P-value
Baseline	122	233.5±7.4	103	230.2±8.1	0.77
1	122	222.4±7.3	103	223.0±8.1	0.90
2	122	217.1±7.6	103	218.0±8.0	0.90
3	122	213.7±8.1	103	215.0±8.0	0.90
4	122	210.0±8.5	103	210.6±8.3	0.96
5	122	203.4±8.6	103	208.2±8.2	0.69
6	115	191.5±9.0	97	203.9±8.7	0.32
7	102	184.0±10.7	92	197.0±9.1	0.32
8	81	183.7±20.0	73	199.3±11.1	0.31
9	64	178.0±11.9	54	199.4±13.2	0.23
10	58	176.1±12.9	47	208.4±15.8	0.11
11	45	181.5±15.4	37	207.9±18.6	0.27
12	32	183.7±20.0	36	200.9±19.1	0.53

**Table IV:** Mean platelet count reductions at different time-points.

Time (days)	Patients (n)	Linezolid group (mean ±SD)	Patients (n)	Glycopeptide group (mean ±SD)	P-value
1	122	11.1±2.5	103	6.4±2.0	0.16
2	122	16.4±3.4	103	11.7±3.4	0.34
3	122	19.8±4.4	103	15.2±4.1	0.46
4	122	23.4±5.3	103	19.6±4.8	0.59
5	122	30.0±5.5	103	22.0±4.9	0.29
6	115	42.0±6.5	97	26.1±5.7	0.07
7	102	51.9±7.0	92	31.9±6.1	0.03
8	81	54.2±8.2	73	30.3±7.8	0.04
9	64	65.6±10.6	54	26.9±9.8	0.01
10	58	65.3±11.2	47	23.9±11.7	0.01
11	45	70.4±13.1	37	13.0±14.1	0.00
12	32	77.9±17.7	36	20.1±12.3	0.00
